# Genetics of rapid eye movement sleep in humans

**DOI:** 10.1038/tp.2015.85

**Published:** 2015-07-07

**Authors:** M Adamczyk, U Ambrosius, S Lietzenmaier, A Wichniak, F Holsboer, E Friess

**Affiliations:** 1 Max Planck Institute of Psychiatry, Munich, Germany

## Abstract

The trait-like nature of electroencephalogram (EEG) is well established. Furthermore, EEG of wake and non-rapid eye movement (non-REM) sleep has been shown to be highly heritable. However, the genetic effects on REM sleep EEG microstructure are as yet unknown. REM sleep is of special interest since animal and human data suggest a connection between REM sleep abnormalities and the pathophysiology of psychiatric and neurological diseases. Here we report the results of a study in monozygotic (MZ) and dizygotic (DZ) twins examining the heritability of REM sleep EEG. We studied the architecture, spectral composition and phasic parameters of REM sleep and identified genetic effects on whole investigated EEG frequency spectrum as well as phasic REM parameters (REM density, REM activity and organization of REMs in bursts). In addition, cluster analysis based on the morphology of the EEG frequency spectrum revealed that the similarity among MZ twins is close to intra-individual stability. The observed strong genetic effects on REM sleep characteristics establish REM sleep as an important source of endophenotypes for psychiatric and neurological diseases.

## Introduction

Trait-like characteristics of human electroencephalographic (EEG) recordings have been extensively studied. Already in the 1930s Travis and Gottlober^1, 2^ reported that brain waves may have an individual pattern. The authors showed that visual comparison of wake EEG signal is sufficient to identify recordings that belong to the same subject. Further studies revealed intra-individual stability of wake EEG frequency spectrum even over years.^3, 4^ Interestingly, this phenomenon was also observed in sleep EEG. Seminal work by Feinberg and colleagues on sleep EEG has established substantial inter-individual variability and high intra-individual stability of both non-rapid eye movement (non-REM) and REM sleep spectral composition.^5,6,7^ Subsequent studies revealed that also after sleep deprivation sleep architecture shows considerable trait inter-individual variability^8^ and non-REM sleep power spectrum remains substantially invariant.^9^ Furthermore, it was shown that the topography of EEG spectral power during non-REM sleep^10^ as well as the EEG spectral pattern during wakefulness,^11^ non-REM and REM sleep^12, 13^ have fingerprint-like characteristics, that is, recordings of each subject cluster together with high accuracy.

The trait-like nature of EEG suggests significant genetic regulation. Indeed, twin studies on the heritability of EEG have reported strong genetic effects on spectral composition during wakefulness^14^ and non-REM sleep.^15, 16^ However, to our knowledge, the genetic regulation of the REM sleep power spectrum has not yet been investigated. Regarding conventional sleep parameters, the duration of slow wave sleep, stage 2 sleep as well as REM density (RD) were shown to be genetically determined, whereas results concerning the duration of REM sleep were inconclusive.^17, 18, 19^

Very high levels of REM sleep until the first year of human life and intense brain activation during that sleep phase suggest that REM sleep strongly contributes to early brain–mind development.^20, 21^ Regarding phasic REM parameters, clustering of REMs increases in infants across the first 4 months of life and reaches a stable level thereafter, whereas the amount of REMs with respect to RD continues to increase.^22^ Becker and Thoman^23^ reported that the number of intense REM periods (so-called REM storms) by the age of 6 months correlates negatively with mental development at 1 year. When comparing healthy young and elderly subjects there is no difference in RD; however, clustering properties of REMs decrease with aging.^24^

The relevance of REM sleep for psychiatric research also derives from its well-established abnormalities in several psychiatric and neurological diseases. The amounts of REM sleep are lower in patients with mental retardation and Alzheimer's disease, whereas patients with narcolepsy and depression show increased REM sleep pressure.^25^ In depression, an increased RD was proposed as a true vulnerability marker: this pathology is present during the acute state of the disease^26^ and also in healthy relatives showing an increased risk for developing an affective disorder.^27^

The trait-like characteristics of sleep EEG, together with the ontogenetic aspects of REM sleep and its alterations in psychiatric and neurological disorders suggest that research on REM sleep is a promising strategy to search for biomarkers and endophenotypes. However, previous twin studies on the genetic influences of REM sleep were restricted to conventional sleep parameters.^17, 18, 19^ We therefore analyzed tonic and phasic REM sleep in a twin study comparing sleep characteristics including sleep architecture, EEG power spectra and phasic REM parameters between healthy monozygotic (MZ) and dizygotic (DZ) twins. To establish a more detailed assessment of phasic REM sleep, in particular, we considered temporal dynamics of REMs throughout the night and the organization of REMs in bursts.^28^

## Materials and methods

### Study sample and design

We analyzed the data of the twin study described by Ambrosius *et al.*^15^ We recruited 35 pairs of MZ and 14 pairs of DZ twins. All twin pairs had been raised together. The twins underwent physical, psychiatric and laboratory examinations to exclude acute and chronic diseases. Prerequisites for inclusion and determination of zygosity are described in detail elsewhere.^15^ Owing to technical reasons (high EEG amplitude differences in consecutive nights), three MZ pairs were excluded. All presented results were obtained from the remaining 32 pairs of MZ twins (mean (s.d.): 23.8 (4.8) years; range: 17–43 years, 16 male pairs, 16 female pairs) and 14 pairs of DZ twins (22.1 (2.7) years; range: 18–26 years, 7 male pairs, 7 female pairs). Fifteen of 32 MZ and 10 of 14 DZ twin pairs were living together at the time of the examination. All investigations were performed at the Max Planck Institute of Psychiatry in Munich. The experimental protocol was approved by the Ethics Committee for Human Experiments of the Bayerische Landesärztekammer (Munich, Germany). Written informed consent was obtained from all participants, after the procedures had been explained. The subjects spent three consecutive nights in our sleep laboratory, where the first night served for adaptation and exclusion of sleep disturbances. Almost all twin partners were recorded at the same time.

### EEG recording

Polysomnographic recordings (Schwarzer, Munich, Germany) were performed according to the international 10–20 electrode system (high-pass filter at 0.53 Hz, low-pass filter at 70 Hz). Electrooculographic (EOG) montage was done according to Rechtschaffen and Kales.^29^ EOG was low- and high-pass-filtered at 30 Hz and 0.095 Hz, respectively. Sleep stages were visually scored in 30-s epochs according to the standard guidelines.^29^ Recordings of the twin partners were scored by the same rater. Fragments with artifacts were excluded from analysis using an automatic procedure (focused on high signal amplitudes, activity in 0.75–3 Hz and 25–45 Hz), followed by visual inspection. We selected the EEG data of the second and third recording night from central derivation C3A2 (results from derivation C4A1 are presented in Supplementary Material).

Fast Fourier transform was performed on the EEG of REM sleep. Power spectra were derived from a 4-s fragment, shifted for 1 s, resulting in a resolution of 0.25 Hz. The resulting spectra were averaged per 30-s epoch. The lowest bins (0.25, 0.5 Hz) were excluded owing to the filtering procedures. For analysis of frequency bands, the power was cumulated across the *δ* (0.75–4.5 Hz), *θ* (4.75–7.75 Hz), *α* (8.0–11.75 Hz), *σ* (12.0–15.75 Hz), *β*_1_ (16.0–25.0 Hz), *β*_2_ (25.25–35.0 Hz), *ϕ* (35.25–45.0 Hz), including a subdivision of the *σ* range into *α*/*σ* (10.0–11.75 Hz), low *σ* (12.0–13.75 Hz) and high *σ* (14.0–15.75 Hz).

### Phasic REM sleep parameters

REMs were detected automatically. In brief, REM is detected if there is a synchronized (within 70 ms) change in EOG potentials of opposite polarity for a time period of minimum 40 ms. In at least one EOG derivation, the potential change must exceed the threshold of 261 μV s^−1^, whereas in the second EOG derivation the potential change must exceed the threshold of 165 μV s^−1^. The automatic algorithm was validated in 12 polysomnographic recordings from seven healthy subjects, which were scored by two expert scorers. Mean epoch-wise correlation for RD scoring between the experts was 0.91 and comparison of automatic scoring with each of the scorers revealed mean correlations of 0.94 and 0.90.

REM activity (RA) was computed with two methods of REMs quantification: first, the number of 3-s mini-epochs containing at least one REM (3sRA), and second, the number of all detected REMs (allRA). We computed RD by averaging RA for specified epochs of REM sleep. RD obtained from allRA is presented as allRD and RD obtained from 3sRA as 3sRD. To investigate the organization of REMs in bursts, we defined REM burst as a sequence of minimum three REMs, where the maximum distance between consecutive REMs is 2 s. We calculated the number of all detected REMs inside REM bursts (RinB), all detected REMs outside REM bursts (RoutB) as well as the percentage of REMs in burst state (RinB%).

### Genetic variance analysis

We investigated MZ and DZ twins to separate the variance of sleep variables into environmental and genetic components according to Christian *et al.*^30, 31^ Briefly, there are two independent estimates of genetic variance: the within-twin-pair estimate (GWT), and the combined within- plus among-twin-pair component estimate (GCT). GWT depends only on mean squares for within-pair variation, whereas GCT depends on mean squares of both within- and among-twin-pair variation. A test of equality of variances (F' test) for MZ and DZ twins determines the selection of genetic variance estimate. We used the GCT test when MZ and DZ variances were equal at <20% probability level (as suggested by the authors). In the other case, the GWT test was used. As a prerequisite for the analysis, each studied variable had to fulfill the assumptions of normal distribution (measured by a nonsignificant goodness-of-fit by the Kolmogorov–Smirnov test) in both twin samples and equal means between twin samples (*T*-test). The influence of covariates (age, sex and cohabitation) was analyzed by multivariate analysis of covariance. Prerequisites were considered to be violated if the appropriate test showed a significant result at the 5% level. Genetic variance analysis was performed on the mean results of two recording nights.

To minimize the effects of possible covariates, we selected a subgroup of MZ twins closely matched for age, gender and cohabitation to DZ twins. Heritability estimations for matched MZ and DZ samples can be found in Supplementary Material.

### ICC analysis

Differences between within-twin pair resemblance and night-to-night stability were illustrated by intraclass correlation coefficients (ICCs). To obtain levels of statistical significance for ICC results, we applied bootstrapping analysis similarly to the study by Tarokh *et al.*^13^ Each sample was recreated by choosing subject values randomly with repetitions up to the same number as in the original set. For each bootstrapped sample ICC was computed. Only positive ICC values of bootstrapped samples were accepted. Bootstrapping was continued until 1000 positive ICC values were reached. For each investigated parameter, we present ICC results of original sample together with upper percentile (*P*=0.01) and median value of bootstrapped data. According to Landis and Koch,^32^ ranges of ICC values were designated as being in slight agreement (from 0 to 0.2), fair agreement (from 0.21 to 0.40), moderate agreement (from 0.41 to 0.60), substantial agreement (from 0.61 to 0.80) and almost perfect agreement (from 0.81 to 1). ICCs estimating within-pair resemblance were performed on mean results of two recording nights.

### Cluster analysis

To investigate the similarity of EEG frequency power spectra with respect to their morphology, we performed hierarchical cluster analysis. Logarithm-transformed power spectra were represented as 178 feature vectors (0.75–45 Hz, 0.25 Hz steps). We chose Pearson's correlation coefficient to obtain information about similarity between vectors. The distance metric used in cluster analysis was 1−Pearson's correlation between vectors. To measure distances between clusters, we applied the shortest distance method that takes the minimal distance of every combination of vectors from both clusters and sets it as the distance between them. Differences between the groups were estimated by comparing similarity coefficient distributions with a non-parametric Wilcoxon rank-sum test.

## Results

### REM sleep architecture

Sample means of averaged over-pairs measures revealed no significant night effects, as well as no significant differences between the twin samples (Supplementary Table S1). Genetic variance analysis of REM sleep architecture identified a significant genetic control of REM sleep duration (see Table 1). However, we observed a significant effect of both age and gender on REM sleep duration. REM sleep was longer in younger subjects and within-pair similarity of REM sleep duration was higher in female twins.

### Phasic REM sleep parameters

The criterion of normal distribution was not fulfilled for RinB, which was therefore logarithm (log) transformed prior to all analyses. Sample means of averaged-over-pairs measures revealed no significant night effects as well as no significant differences between the twin samples (Supplementary Table S2). None of the covariates significantly affected the results. Genetic variance analysis revealed a significant genetic control for mean RD throughout the whole night (allRD and 3sRD), including the 1st and 2nd sleep cycle and the 2nd third of the night. Genetic effects on RD in the 3rd sleep cycle and the other thirds of the night were marginally significant. Furthermore, we identified a significant genetic influence on whole-night RA (allRA and 3sRA), RinB and RinB% (see Table 1). According to the Landis and Koch^32^ benchmark, ICCs of all phasic REM parameters considered over the whole night for consecutive nights were at least substantial. However, when analyzing RD in fragments of night sleep, night-to-night stability was comparably low and showed considerable variation between MZ and DZ sets. Differences between twin groups in the stability of RD for the 1st and 3rd cycle and the last third of the night were considerable; therefore, genetic variance analysis estimations in these fragments should be treated with caution. RoutB was the only phasic parameter measured over the whole night for which we found no genetic effects. Here, a substantial within-pair similarity was observed in both DZ and MZ twins.

### Spectral composition of REM sleep

The criterion of normal distribution was not fulfilled for spectral power in many EEG frequency bins. Therefore, all spectral data were log transformed prior to analysis. Genetic variance analysis was not applicable for the 1-Hz bin, 16–21 Hz bins nor for *β*_1_ frequency band, as sample means of averaged over-pairs measurements revealed significant differences between the twin samples (Supplementary Tables S3 and S4). We identified a genetic influence on EEG power in all remaining frequency bins (2–15 Hz, 22–45 Hz; Supplementary Table S5) and bands (*δ* band to high*-σ* band, *β*_2_ band to *ϕ* band, see Table 1). However, gender, as a covariate, had a significant effect on spectral power values (higher power values in *δ*, *θ*, *β*_2_ and *ϕ* bands or 1–7 Hz and 27–45 Hz bins in female subjects).

**Figure 1:**
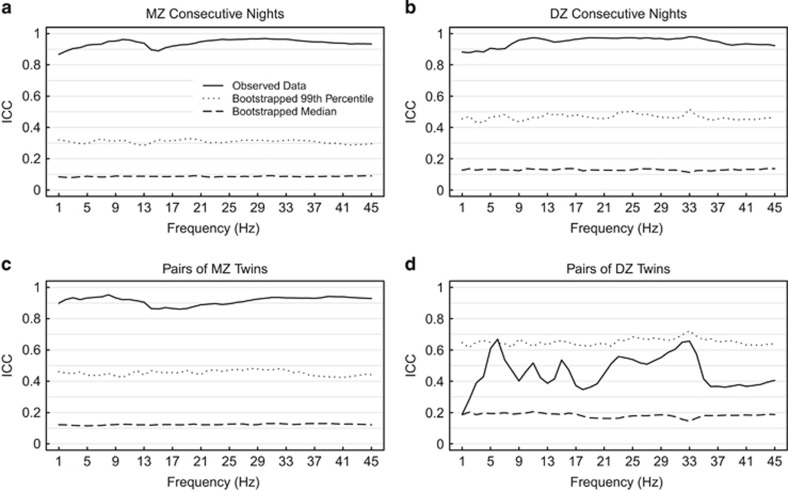
ICCs of REM sleep frequency bins. In each plot the solid line represents the observed real data, dotted line represents the upper percentile of bootstrapped values and dashed line represents the median of bootstrapped values. (**a**) Consecutive nights of each subject in the MZ set (*n*=64); (**b**) consecutive nights of each subject in the DZ set (*n*=28); (**c**) pairs of MZ twins (each subject represented by a two-night mean, *n*=32); (**d**) pairs of DZ twins (each subject represented by a two-night mean, *n*=14). On average, the upper percentile and the median of bootstrapped values differ between groups, which is the outcome of different sample sizes. DZ, dizygotic; MZ, monozygotic; ICC, intraclass correlation coefficient; REM, rapid eye movement.

ICC results with respect to EEG frequency bins are illustrated in Figure 1. The mean ICC for all EEG frequency bins was 0.91 in the MZ twins and 0.45 in the DZ twins. In contrast, the mean ICCs for night-to-night stability were similar between MZ (0.94) and DZ (0.92) twins. Bin-wise ICC values for MZ twins and night-to-night stability in both MZ and DZ groups were 'almost perfect'. ICC results for DZ twins were irregular throughout the frequency spectrum showing no within-pair similarity above the significance threshold (*P*=0.01, Figure 1d). This effect was probably influenced by the smaller size of the DZ twins sample. As the consecutive nights stability within DZ set was 'almost perfect', it is unlikely that the low within-pair similarity is caused by the bad quality of sleep recordings.

**Figure 2:**
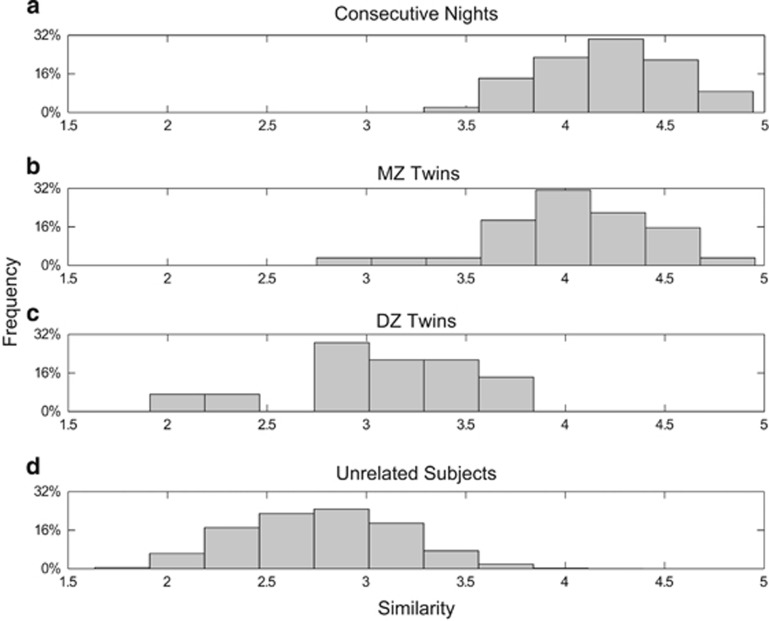
Distribution of Fisher's *z-*transformed (*z*) Pearson's correlations for power spectra in rapid eye movement sleep. Each power spectrum was a 178-feature vector (0.75–45 Hz, 0.25 Hz bins). (**a**) Consecutive nights of each subject (*n*=92); (**b**) pairs of MZ twins (each subject represented by a two-night mean, *n*=32); (**c**) pairs of DZ twins (each subject represented by a two-night mean, *n*=14); (**d**) unrelated subjects (*n*=16 560). If there is no similarity *z*=0; if there is perfect similarity *z*=infinity. DZ, dizygotic; MZ, monozygotic.

Figure 2 illustrates the distribution of Fisher‘s *z*-transformed correlation values between power spectra within different groups. The mean (s.d.) of Fisher's *z*-transformed correlation values was 4.09 (0.29) between consecutive nights, 3.94 (0.35) between MZ twins, 3.10 (0.42) between DZ twins and 2.77 (0.39) between unrelated subjects. Pairwise comparisons of similarity distributions between these groups revealed only a marginal difference between intra-individual and MZ twins similarity (*P*=0.0821, Wilcoxon rank-sum test). The means of all other groups were significantly different when compared pairwise (*P*=0.0015 when comparing DZ twins with inter-individual similarity and *P*<0.0001 for all other comparisons).

**Figure 3:**
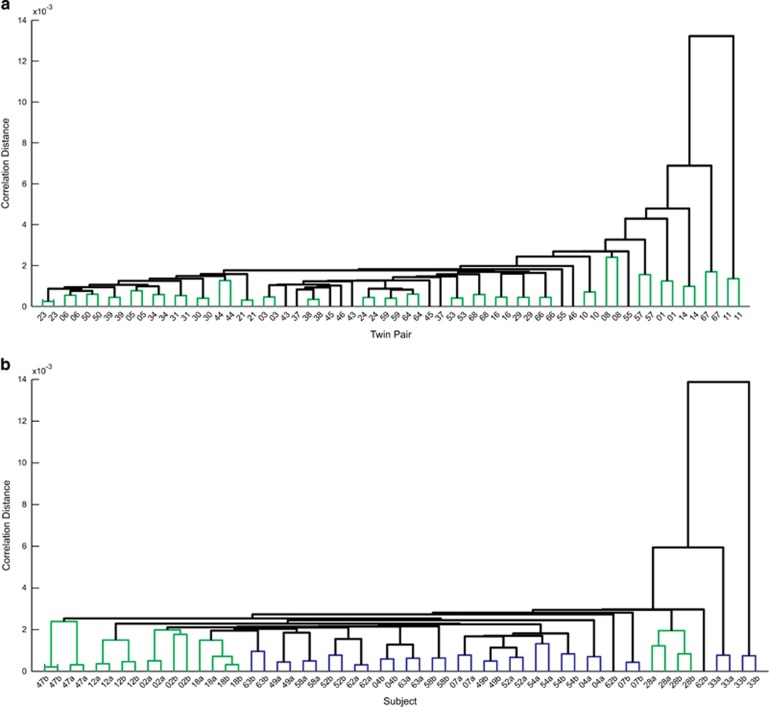
Dendrograms of cluster analysis based on distances between power spectra for rapid eye movement sleep in (**a**) MZ and (**b**) DZ twins. In **a**, each subject is represented by a two-night mean power spectrum, and subjects with the same number represent the same MZ pair. In **b**, each subject is represented by two separate nights and the subjects' IDs consist of a number (defines the DZ pair) and a character (defines a twin within the pair). In both **a** and **b**, the subjects IDs are on the *x* axis; the distance between clusters is on the *y* axis. Each power spectrum was a 178-feature vector (0.75–45 Hz). Distance metric was 1−Pearson's correlation between vectors. Green clusters depict MZ or DZ pairs that clustered together. Blue clusters depict DZ twins whose consecutive nights clustered together but not with related DZ twins. DZ, dizygotic; ID, identity; MZ, monozygotic.

We performed cluster analysis on mean EEG spectra measured from two recording nights, as well as using both nights individually (Figure 3). When each night was treated individually, we assumed that the two nights for the same subject clustered if the distance from each other was smaller than the distance from any unrelated subject. We assumed that a twin pair clustered if the distance between the twins for any combination of their nights was smaller than the distance between the twins and any unrelated subject.

Analysis of the mean spectra of two nights revealed that 27 of 32 twin pairs clustered within the MZ set, whereas only 4 of 14 DZ pairs clustered. Clustering performed on individual nights showed similar results. The same pairs clustered within the MZ set and one additional pair clustered within the DZ set. With respect to consecutive nights, 60 of 64 subjects clustered within the MZ set and 27 of 28 within the DZ set.

**Figure 4:**
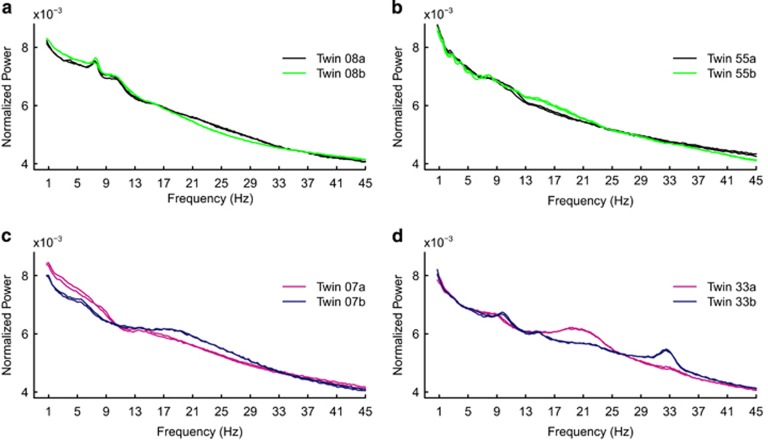
Logarithm-transformed and normalized power spectra for REM sleep of the two most dissimilar (according to Pearson's correlation between power spectra) monozygotic twin pairs (**a**, **b**) and the two most dissimilar dizygotic twin pairs (**c**, **d**). Consecutive nights of all presented twins clustered correctly. Each plot consists of four power spectra from one twin pair. REM spectra of twin siblings are shown in different colors; consecutive nights in each individual have the same color. REM, rapid eye movement.

To investigate whether the EEG power spectrum at higher frequencies yields any additional information, we repeated the clustering experiment after separating low from high frequencies. The increase in data size makes the clustering procedure more demanding (higher probability for similar spectra to occur in unrelated subjects by chance). Therefore, we analyzed all 184 nights from the combined MZ and DZ sets. Cluster analysis performed on 0.75–45 Hz frequencies revealed that 89.1% of consecutive nights, 81.3% of MZ pairs and 14.3% of DZ pairs clustered. When frequencies were limited to 20 Hz, we observed that only 73.9% of consecutive nights clustered. The percentage of MZ and DZ pairs that clustered did not change, although the groups of MZ pairs that clustered were not the same. The least similar power spectra within a twin pair are presented for both MZ and DZ twins in Figure 4. The MZ twin pair illustrated in Figure 4b clustered when the spectrum was limited to 20 Hz and failed to cluster when it was extended to 45 Hz.

## Discussion

In this study, we compared REM sleep architecture, REM EEG spectral power, shape of power spectrum, amount and structural organization of REMs between a group of 32 healthy MZ and 14 DZ same-gender twins. The genetic variance analysis was used to estimate the genetic effects. In addition, within-pair similarity and night-to-night stability of given parameters were illustrated by ICCs and cluster analysis.

We identified a strong genetic determination of the absolute and relative amount and the structural organization of REMs, with the exception of the REMs occurring outside REM bursts. The genetic effects on REMs were stable and independent of the definition of given phasic REM parameters. Furthermore, we observed a substantial genetic influence on REM EEG power spectrum (*δ* to high *σ*, *β*_2_ to *γ* band). We also observed significant differences in within-pair similarity of REM EEG spectra morphology between the twin groups. Interestingly, a strong genetic influence and high night-to-night stability were also detected in high EEG frequencies. Restricting the frequency range to 20 Hz resulted in a drop in clustering of consecutive nights, whereas clustering rates of twin pairs did not change. Here, single individuals exhibited characteristic features in high frequencies (see Figure 4d). None of the estimates of genetic variance in REM EEG differed between derivations C3A2 and C4A1 (see Supplementary Material). Due to possible effects of the covariates, analyses were repeated with a subgroup of MZ twins closely matched for age, gender and cohabitation to DZ twins. The corresponding results confirmed our findings in the total twin sample (see Supplementary Material). In addition, we found significant genetic influence for the remaining seven frequency bins (1 Hz, 16–21 Hz) and *β*_1_ frequency band of REM sleep EEG.

The trait-like nature of EEG is well established. A number of studies emphasized the individuality and stability of EEG features during both wakefulness and sleep in humans.^1, 2, 3, 4, 5, 6, 7, 8, 9, 10, 11, 12, 13^ In our study we also observed a strong trait-like character of both REM sleep spectral composition and phasic REM sleep parameters considered over the whole night. The comparison of night-to-night stability with the similarity between twins revealed that the similarity between MZ twins always closely resembled and sometimes exceeded intra-individual stability. In DZ twins, however, within-pair similarity was clearly lower than their night-to-night stability. Here, significant within-pair similarity was observed only for REMs occurring outside REM bursts. The REM sleep parameters with the best night-to-night stability showed the strongest genetic component. The observed coincidence of night-to-night stability and genetic effects applied to all investigated tonic and phasic REM sleep parameters. This phenomenon has already been reported in previous twin studies with smaller samples.^17, 18, 19^

Cluster analysis of REM EEG power spectra was performed according to previous reports on trait-like characteristics of sleep EEG.^11, 12, 13^ We found that clustering properties of REM EEG spectra significantly differed between MZ and DZ twins (Figure 2). Again, the distribution of MZ similarities was close to their intra-individual stability, whereas the distribution of DZ similarities resembled results in unrelated subjects. For further illustration, Figure 4 shows that discrepancies between the most dissimilar EEG spectra of MZ twins are very small when compared with the most dissimilar spectra of DZ twins.

All estimations of genetic effects considered absolute EEG power values where part of the observed genetic variance could be an outcome of skull and scalp thickness. Both variables influence EEG power and are most likely heritable. However, cluster analysis based on EEG morphology, which is independent of absolute signal amplitude, confirmed strong heritability of EEG in REM sleep, independently from skull and scalp thickness.

In summary, the present results support a substantial genetic determination of both tonic and phasic REM sleep parameters and complement previous findings of a high genetic determination of the non-REM sleep power spectrum.^15, 16^ Our results highlight the potential of REM sleep abnormalities as a source of clinically relevant biomarkers for psychiatric and neurological diseases.^25^ For example, recent research on REM sleep EEG indicates that prefrontal *θ* cordance could provide a biomarker for antidepressant treatment outcome.^33^ In the present study, we were able to demonstrate strong genetic effects on REM EEG frequencies up to 45 Hz. In view of the given interference of muscle artifacts with EEG analysis, investigating REM sleep, which is characterized by muscle atonia, may offer a better alternative to identify disease-specific differences in higher EEG frequencies.

## Figures and Tables

**Table 1:** Genetic variance analysis and intraclass correlation coefficients for REM sleep architecture, phasic REM parameters and EEG frequency bands in REM sleep

*Variable*	P	*GWT vs GCT*	*ICC MZ*	*ICC DZ*	*ICC MZ cn*	*ICC DZ cn*
*REM sleep architecture*						
REM sleep duration	0.0462	GWT	0.71 (0.45, 0.12)	0.55 (0.65, 0.19)	0.46 (0.35, 0.09)	0.56 (0.43, 0.13)
REM sleep latency	0.4948	GWT	0.46 (0.47, 0.12)	0.50 (0.72, 0.15)	0.27 (0.33, 0.08)	0.57 (0.53, 0.11)

*Phasic REM parameters*						
allRA all night	0.0020	GWT	0.88 (0.57, 0.11)	0.45 (0.66, 0.19)	0.91 (0.38, 0.08)	0.72 (0.48, 0.13)
3sRA all night	0.0054	GWT	0.85 (0.49, 0.11)	0.50 (0.63, 0.19)	0.88 (0.36, 0.08)	0.78 (0.45, 0.13)
allRD all night	0.0051	GWT	0.84 (0.53, 0.11)	0.33 (0.61, 0.18)	0.89 (0.38, 0.08)	0.66 (0.48, 0.13)
3sRD all night	0.0027	GWT	0.83 (0.47, 0.12)	0.31 (0.66, 0.19)	0.85 (0.35, 0.09)	0.74 (0.48, 0.13)
3sRD 1st cycle	0.0036	GCT	0.58 (0.50, 0.12)	−0.16 (0.66, 0.18)	0.49 (0.34, 0.08)	0.07 (0.51, 0.13)
3sRD 2nd cycle	0.0096	GWT	0.70 (0.44, 0.12)	0.04 (0.61, 0.20)	0.41 (0.33, 0.08)	0.60 (0.47, 0.14)
3sRD 3rd cycle	0.0774	GWT	0.68 (0.49, 0.11)	0.22 (0.67, 0.18)	0.71 (0.32, 0.08)	0.38 (0.50, 0.13)
3sRD 1st third	0.0965	GWT	0.60 (0.48, 0.12)	0.21 (0.68, 0.19)	0.47 (0.33, 0.08)	0.32 (0.49, 0.13)
3sRD 2nd third	0.0041	GCT	0.80 (0.50, 0.11)	0.19 (0.62, 0.18)	0.73 (0.32, 0.09)	0.69 (0.46, 0.13)
3sRD 3rd third	0.0762	GWT	0.71 (0.47, 0.11)	0.31 (0.65, 0.20)	0.68 (0.33, 0.08)	0.44 (0.47, 0.13)
RinB all night	0.0002	GWT	0.75 (0.46, 0.12)	0.25 (0.66, 0.18)	0.80 (0.31, 0.08)	0.87 (0.49, 0.13)
RoutB all night	0.2467	GWT	0.71 (0.46, 0.12)	0.66 (0.62, 0.17)	0.80 (0.33, 0.09)	0.69 (0.47, 0.13)
RinB% all night	0.0002	GWT	0.70 (0.45, 0.12)	0.13 (0.70, 0.19)	0.64 (0.29, 0.09)	0.81 (0.48, 0.13)

*Frequency bands in REM sleep*						
Δ	0.0008	GCT	0.92 (0.45, 0.12)	0.27 (0.59, 0.18)	0.89 (0.31, 0.09)	0.87 (0.46, 0.13)
Θ	0.0001	GWT	0.93 (0.44, 0.13)	0.51 (0.66, 0.19)	0.92 (0.33, 0.09)	0.90 (0.46, 0.13)
A	<0.0001	GWT	0.91 (0.45, 0.13)	0.40 (0.69, 0.19)	0.95 (0.34, 0.09)	0.95 (0.49, 0.13)
Σ	0.0001	GWT	0.89 (0.45, 0.12)	0.45 (0.64, 0.19)	0.91 (0.32, 0.09)	0.92 (0.50, 0.13)
*α*/*σ*	0.0002	GWT	0.90 (0.41, 0.12)	0.52 (0.62, 0.19)	0.94 (0.33, 0.08)	0.95 (0.48, 0.13)
Low *σ*	<0.0001	GWT	0.89 (0.47, 0.12)	0.41 (0.62, 0.19)	0.92 (0.31, 0.09)	0.93 (0.46, 0.13)
High *σ*	0.0004	GWT	0.89 (0.41, 0.12)	0.53 (0.63, 0.18)	0.90 (0.32, 0.09)	0.92 (0.51, 0.13)
*β*_1_^a^	—	—	0.89 (0.47, 0.12)	0.41 (0.76, 0.17)	0.95 (0.31, 0.09)	0.97 (0.59, 0.12)
*β*_2_	0.0008	GWT	0.92 (0.45, 0.12)	0.62 (0.71, 0.17)	0.97 (0.32, 0.09)	0.96 (0.53, 0.12)
*Φ*	<0.0001	GWT	0.92 (0.46, 0.12)	0.40 (0.61, 0.19)	0.94 (0.33, 0.09)	0.87 (0.47, 0.13)

Abbreviations: allRD, allRA divided by the number of REM sleep epochs; allRA, the number of all detected REMs; DZ, dizygotic; ICC, intraclass correlation coefficient; ICC MZ, ICC of MZ twin pairs; ICC DZ, ICC of DZ twin pairs; ICC MZ cn, ICCs of consecutive nights for each subject in the MZ group; ICC DZ cn, ICCs of consecutive nights for each subject in the DZ group; MZ, monozygotic; RA, REM activity; RD, REM density; REM, rapid eye movement; RinB, the number of all detected REMs inside REM bursts; RinB%, percentage of REMs inside REM bursts; RoutB, the number of all detected REMs outside REM bursts; 3sRA, the number of 3-s mini-epochs containing at least one REM; 3sRD, 3sRA divided by the number of REM sleep epochs.

Results of genetic variance analysis, type of estimate applied (GCT: combined among- and within-twin pair component estimate, GWT: within-pair estimate) and ICCs. ICC results include: original sample ICC (upper percentile of bootstrapped data and median of bootstrapped data).

a Analysis of variance not applicable (significant differences between the means in the DZ and MZ twin sets).
