# Supplementary material for: Genetics of rapid eye movement sleep in humans
Source: Transl Psychiatry. 2015 Jul 7;5(7):e598–. doi: 10.1038/tp.2015.85 (PMC5068721; doi:10.1038/tp.2015.85)
Supplement: Supplementary Tables [file tp201585x1.doc]

Genetics of Rapid Eye Movement Sleep in Humans: Supplementary Material

Marek Adamczyk (MSc), Urte Ambrosius (MD), Sonja Lietzenmaier (MD), Adam Wichniak (MD), Florian Holsboer (Ph.D., M.D.), Elisabeth Friess (MD)

**Supplementary Table S1:** Sleep Architecture Parameters Averaged Over Pairs

|  | DZ n = 14 | | | MZ n = 32 | | |
| --- | --- | --- | --- | --- | --- | --- |
|  | *Night 2* | *Night 3* | *2 nights mean* | *Night 2* | *Night 3* | *2 nights mean* |
| TST | 419.33±6.27 | 423.64±5.65 | 421.49±5.73 | 420.89±4.01 | 417.51±3.94 | 419.20±3.39 |
| SPT | 462.75±4.31 | 466.57±3.23 | 464.66±3.55 | 469.26±2.23 | 469.85±1.72 | 469.56±1.62 |
| SEI | 0.94±0.01 | 0.95±0.01 | 0.95±0.01 | 0.94±0.01 | 0.94±0.01 | 0.94±0.01 |
| SOL | 30.05±4.66 | 27.23±3.55 | 28.64±3.94 | 23.41±2.27 | 23.14±1.56 | 23.27±1.61 |
| non-REM | 324.21±6.17 | 324.89±4.50 | 324.55±5.09 | 321.54±3.16 | 318.48±3.64 | 320.01±2.98 |
| REM | 95.12±4.09 | 98.75±5.07 | 96.93±4.24 | 99.34±3.22 | 99.03±2.55 | 99.18±2.58 |
| RSL | 99.42±9.37 | 99.08±9.19 | 99.25±8.81 | 104.13±5.21 | 99.72±4.40 | 101.92±4.28 |

Group mean ± SEM of sleep characteristics in minutes. TST: Total sleep time, SPT: Sleep period time, SEI: sleep efficiency index, SOL: sleep onset latency, REM: rapid eye movement, non-REM: non-REM sleep duration, REM: REM sleep duration, RSL: REM sleep latency, DZ: dizygotic twins, MZ: monozygotic twins.

**Supplementary Table S2:** Phasic REM Parameters Averaged Over Pairs

|  | DZ n = 14 | | | MZ n = 32 | | |
| --- | --- | --- | --- | --- | --- | --- |
|  | *Night 2* | *Night 3* | *2 nights mean* | *Night 2* | *Night 3* | *2 nights mean* |
| allRD all night | 4.27±0.53 | 4.26±0.40 | 4.26±0.45 | 3.58±0.41 | 3.64±0.42 | 3.61±0.41 |
| 3sRD all night | 2.06±0.19 | 2.11±0.15 | 2.08±0.16 | 1.81±0.14 | 1.85±0.15 | 1.83±0.14 |
| 3sRD 1^st^ cycle | 1.07±0.14 | 1.20±0.15 | 1.13±0.10 | 0.98±0.13 | 0.97±0.10 | 0.97±0.10 |
| 3sRD 2^nd^ cycle | 1.85±0.19 | 1.73±0.17 | 1.79±0.16 | 1.53±0.14 | 1.64±0.18 | 1.58±0.14 |
| 3sRD 3^rd^ cycle | 1.92±0.19 | 2.11±0.22 | 2.01±0.19 | 1.82±0.18 | 1.76±0.17 | 1.79±0.17 |
| 3sRD 1^st^ third | 1.12± 0.20 | 1.11±0.15 | 1.11±0.15 | 1.00±0.12 | 1.15±0.12 | 1.07±0.12 |
| 3sRD 2^nd^ third | 1.90±0.18 | 2.03±0.19 | 1.97±0.17 | 1.81±0.19 | 1.75±0.17 | 1.78±0.17 |
| 3sRD 3^rd^ third | 2.39±0.22 | 2.34±0.16 | 2.37±0.17 | 2.01±0.15 | 2.11±0.16 | 2.06±0.15 |
| allRA all night | 831.85±118.58 | 860.03±93.90 | 845.94±100.30 | 710.07±95.80 | 712.84±86.87 | 711.46±90.13 |
| 3sRA all night | 402.14±47.36 | 425.00±39.38 | 413.57±40.95 | 357.92±33.71 | 363.17±31.39 | 360.54±31.95 |
| RinB all night* | 2.63±0.09 | 2.69±0.07 | 2.67±0.08 | 2.56±0.05 | 2.56±0.05 | 2.57±0.05 |
| RoutB all night | 214.71±18.38 | 234.67±18.62 | 224.69±17.44 | 206.92±11.79 | 214.64±10.89 | 210.78±10.97 |
| RinB% all night | 0.66±0.02 | 0.67±0.02 | 0.67±0.03 | 0.64±0.01 | 0.63±0.02 | 0.64±0.02 |

Group mean ± SEM. REM: rapid eye movement, DZ: dizygotic twins, MZ: monozygotic twins. RA: REM activity, RD: REM density, allRA: the number of all detected REMs, 3sRA: the number of 3-sec mini-epochs containing at least one REM, allRD: allRA divided by the number of REM sleep epochs, 3sRD: 3sRA divided by the number of REM sleep epochs, RinB: the number of all detected REMs inside REM bursts, RoutB: the number of all detected REMs outside REM bursts, RinB%: percentage of REMs in burst state.

* Logarithm (base 10) transformed data.

Derivation C3A2:

**Supplementary Table S3:** EEG Frequency Bands in REM Sleep Averaged Over Pairs from C3A2 EEG Derivation

|  | DZ n = 14 | | | MZ n = 32 | | |
| --- | --- | --- | --- | --- | --- | --- |
|  | *Night 2* | *Night 3* | *2 nights mean* | *Night 2* | *Night 3* | *2 nights mean* |
| δ | 2.10±0.03 | 2.09±0.03 | 2.10±0.03 | 2.03±0.03 | 2.02±0.03 | 2.02±0.03 |
| θ | 1.48±0.04 | 1.47±0.04 | 1.48±0.04 | 1.47±0.03 | 1.46±0.03 | 1.47±0.03 |
| α | 1.22±0.04 | 1.21±0.04 | 1.21±0.04 | 1.15±0.03 | 1.14±0.03 | 1.15±0.03 |
| σ | 0.79±0.03 | 0.77±0.04 | 0.78±0.03 | 0.70±0.03 | 0.68±0.03 | 0.69±0.03 |
| α/σ | 0.77±0.04 | 0.75±0.04 | 0.76±0.04 | 0.68±0.03 | 0.67±0.03 | 0.68±0.03 |
| low σ | 0.54±0.03 | 0.52±0.04 | 0.53±0.03 | 0.45±0.03 | 0.44±0.03 | 0.45±0.03 |
| high σ | 0.41±0.03 | 0.39±0.04 | 0.40±0.03 | 0.33±0.03 | 0.31±0.03 | 0.32±0.03 |
| β1* | 0.81±0.04 | 0.79±0.04 | 0.80±0.04 | 0.69±0.03 | 0.68±0.03 | 0.69±0.03 |
| β2 | 0.23±0.04 | 0.22±0.04 | 0.22±0.04 | 0.16±0.03 | 0.16±0.03 | 0.16±0.03 |
| φ | -0.25±0.02 | -0.26±0.02 | -0.25±0.02 | -0.31±0.02 | -0.30±0.02 | -0.31±0.02 |

Group mean ± SEM. Logarithm (base 10) transformed mean power densities from C3A2 EEG derivation (given in μV²).

EEG: electroencephalogram, REM: rapid eye movement, DZ: dizygotic twins, MZ: monozygotic twins.

* DZ and MZ means are not equal at the 5% level.

**Supplementary Table S4:** EEG 1-Hz Frequency Bins in REM Sleep Averaged Over Pairs from C3A2 EEG Derivation

|  | DZ n = 14 | | | MZ n = 32 | | |
| --- | --- | --- | --- | --- | --- | --- |
|  | *Night 2* | *Night 3* | *2 nights mean* | *Night 2* | *Night 3* | *2 nights mean* |
| 1 Hz* | 1.46±0.03 | 1.47±0.03 | 1.47±0.03 | 1.36±0.03 | 1.36±0.03 | 1.36±0.03 |
| 2 Hz | 1.63±0.03 | 1.62±0.03 | 1.62±0.03 | 1.54±0.03 | 1.53±0.03 | 1.54±0.03 |
| 3 Hz | 1.45±0.03 | 1.43±0.03 | 1.44±0.03 | 1.39±0.03 | 1.38±0.03 | 1.39±0.03 |
| 4 Hz | 1.27±0.03 | 1.25±0.03 | 1.27±0.03 | 1.23±0.03 | 1.21±0.03 | 1.22±0.03 |
| 5 Hz | 1.14±0.04 | 1.12±0.03 | 1.13±0.04 | 1.10±0.03 | 1.08±0.03 | 1.09±0.03 |
| 6 Hz | 1.03±0.04 | 1.02±0.04 | 1.03±0.04 | 1.01±0.03 | 1.00±0.03 | 1.01±0.03 |
| 7 Hz | 0.91±0.04 | 0.89±0.03 | 0.90±0.03 | 0.90±0.03 | 0.90±0.03 | 0.90±0.03 |
| 8 Hz | 0.82±0.04 | 0.80±0.04 | 0.81±0.04 | 0.81±0.04 | 0.80±0.04 | 0.81±0.04 |
| 9 Hz | 0.74±0.05 | 0.73±0.05 | 0.74±0.04 | 0.69±0.03 | 0.68±0.04 | 0.69±0.03 |
| 10 Hz | 0.64±0.05 | 0.63±0.05 | 0.64±0.05 | 0.56±0.03 | 0.56±0.03 | 0.56±0.03 |
| 11 Hz | 0.49±0.04 | 0.48±0.04 | 0.49±0.04 | 0.40±0.03 | 0.40±0.03 | 0.40±0.03 |
| 12 Hz | 0.35±0.04 | 0.34±0.04 | 0.34±0.04 | 0.27±0.03 | 0.26±0.03 | 0.26±0.03 |
| 13 Hz | 0.25±0.03 | 0.23±0.04 | 0.24±0.03 | 0.16±0.03 | 0.15±0.03 | 0.16±0.03 |
| 14 Hz | 0.18±0.03 | 0.16±0.03 | 0.17±0.03 | 0.10±0.03 | 0.08±0.03 | 0.09±0.03 |
| 15 Hz | 0.15±0.03 | 0.12±0.04 | 0.14±0.04 | 0.05±0.03 | 0.03±0.03 | 0.04±0.03 |
| 16 Hz* | 0.08±0.03 | 0.06±0.04 | 0.07±0.04 | -0.03±0.03 | -0.04±0.03 | -0.03±0.03 |
| 17 Hz* | 0.02±0.03 | -0.00±0.04 | 0.01±0.04 | -0.10±0.03 | -0.11±0.03 | -0.11±0.02 |
| 18 Hz* | -0.03±0.04 | -0.05±0.04 | -0.04±0.04 | -0.15±0.03 | -0.16±0.03 | -0.16±0.03 |
| 19 Hz* | -0.07±0.04 | -0.09±0.05 | -0.08±0.04 | -0.20±0.03 | -0.21±0.03 | -0.20±0.03 |
| 20 Hz* | -0.11±0.04 | -0.13±0.05 | -0.12±0.05 | -0.24±0.03 | -0.25±0.03 | -0.25±0.03 |
| 21 Hz* | -0.16±0.04 | -0.18±0.05 | -0.17±0.05 | -0.29±0.03 | -0.30±0.03 | -0.30±0.03 |
| 22 Hz | -0.24±0.05 | -0.26±0.05 | -0.25±0.05 | -0.35±0.03 | -0.36±0.03 | -0.35±0.03 |
| 23 Hz | -0.31±0.05 | -0.33±0.05 | -0.32±0.05 | -0.41±0.03 | -0.42±0.03 | -0.42±0.03 |
| 24 Hz | -0.39±0.04 | -0.41±0.05 | -0.40±0.04 | -0.49±0.03 | -0.49±0.03 | -0.49±0.03 |
| 25 Hz | -0.47±0.04 | -0.48±0.04 | -0.48±0.04 | -0.55±0.03 | -0.55±0.03 | -0.55±0.03 |
| 26 Hz | -0.54±0.04 | -0.55±0.04 | -0.55±0.04 | -0.62±0.03 | -0.62±0.03 | -0.62±0.03 |
| 27 Hz | -0.61±0.04 | -0.62±0.04 | -0.61±0.04 | -0.68±0.03 | -0.68±0.03 | -0.68±0.03 |
| 28 Hz | -0.67±0.03 | -0.68±0.04 | -0.67±0.04 | -0.74±0.03 | -0.73±0.03 | -0.73±0.03 |
| 29 Hz | -0.73±0.03 | -0.74±0.04 | -0.74±0.04 | -0.79±0.03 | -0.79±0.03 | -0.79±0.03 |
| 30 Hz | -0.78±0.03 | -0.80±0.04 | -0.79±0.04 | -0.85±0.03 | -0.84±0.03 | -0.84±0.03 |
| 31 Hz | -0.84±0.03 | -0.86±0.04 | -0.85±0.04 | -0.90±0.03 | -0.90±0.03 | -0.90±0.03 |
| 32 Hz | -0.89±0.04 | -0.91±0.04 | -0.90±0.04 | -0.95±0.03 | -0.95±0.03 | -0.95±0.03 |
| 33 Hz | -0.93±0.04 | -0.94±0.05 | -0.93±0.05 | -1.01±0.03 | -1.00±0.03 | -1.01±0.03 |
| 34 Hz | -0.97±0.04 | -0.99±0.05 | -0.98±0.04 | -1.06±0.03 | -1.05±0.03 | -1.05±0.03 |
| 35 Hz | -1.03±0.03 | -1.04±0.04 | -1.03±0.03 | -1.10±0.03 | -1.10±0.03 | -1.10±0.03 |
| 36 Hz | -1.08±0.03 | -1.09±0.03 | -1.08±0.03 | -1.15±0.03 | -1.14±0.02 | -1.15±0.02 |
| 37 Hz | -1.13±0.03 | -1.14±0.03 | -1.13±0.03 | -1.19±0.02 | -1.19±0.02 | -1.19±0.02 |
| 38 Hz | -1.17±0.03 | -1.19±0.03 | -1.18±0.02 | -1.23±0.02 | -1.23±0.02 | -1.23±0.02 |
| 39 Hz | -1.21±0.02 | -1.23±0.03 | -1.22±0.02 | -1.27±0.02 | -1.27±0.02 | -1.27±0.02 |
| 40 Hz | -1.25±0.02 | -1.26±0.02 | -1.26±0.02 | -1.31±0.02 | -1.30±0.02 | -1.31±0.02 |
| 41 Hz | -1.29±0.02 | -1.30±0.02 | -1.29±0.02 | -1.34±0.02 | -1.34±0.02 | -1.34±0.02 |
| 42 Hz | -1.32±0.02 | -1.33±0.02 | -1.33±0.02 | -1.38±0.02 | -1.37±0.02 | -1.37±0.02 |
| 43 Hz | -1.36±0.02 | -1.36±0.02 | -1.36±0.02 | -1.41±0.02 | -1.41±0.02 | -1.41±0.02 |
| 44 Hz | -1.39±0.02 | -1.40±0.02 | -1.39±0.02 | -1.45±0.02 | -1.44±0.02 | -1.44±0.02 |
| 45 Hz | -1.42±0.02 | -1.43±0.02 | -1.43±0.02 | -1.48±0.02 | -1.47±0.02 | -1.48±0.02 |

Group mean ± SEM. Logarithm (base 10) transformed mean power densities from C3A2 EEG derivation (given in μV²). EEG: electroencephalogram, REM: rapid eye movement, DZ: dizygotic twins, MZ: monozygotic twins.

* DZ and MZ means are not equal at the 5% level.

**Supplementary Table S5: Genetic Variance Analysis on EEG 1-Hz Frequency Bins in REM Sleep from C3A2 EEG Derivation**

| Variable | *P* | GWT vs GCT |
| --- | --- | --- |
| 1 Hz* | - | - |
| 2 Hz | .0012 | GCT |
| 3 Hz | .0012 | GCT |
| 4 Hz | .0013 | GCT |
| 5 Hz | .0006 | GWT |
| 6 Hz | .0009 | GWT |
| 7 Hz | .0055 | GCT |
| 8 Hz | .0015 | GCT |
| 9 Hz | <.0001 | GWT |
| 10 Hz | <.0001 | GWT |
| 11 Hz | .0002 | GWT |
| 12 Hz | .0001 | GWT |
| 13 Hz | <.0001 | GWT |
| 14 Hz | .0002 | GWT |
| 15 Hz | .0007 | GWT |
| 16 Hz* | - | - |
| 17 Hz* | - | - |
| 18 Hz* | - | - |
| 19 Hz* | - | - |
| 20 Hz* | - | - |
| 21 Hz* | - | - |
| 22 Hz | <.0001 | GWT |
| 23 Hz | <.0001 | GWT |
| 24 Hz | .0001 | GWT |
| 25 Hz | .0003 | GWT |
| 26 Hz | .0004 | GWT |
| 27 Hz | .0004 | GWT |
| 28 Hz | .0007 | GWT |
| 29 Hz | .0008 | GWT |
| 30 Hz | .0018 | GWT |
| 31 Hz | .0015 | GWT |
| 32 Hz | .0008 | GWT |
| 33 Hz | <.0001 | GWT |
| 34 Hz | <.0001 | GWT |
| 35 Hz | <.0001 | GWT |
| 36 Hz | <.0001 | GWT |
| 37 Hz | .0003 | GWT |
| 38 Hz | .0045 | GCT |
| 39 Hz | .0039 | GCT |
| 40 Hz | <.0001 | GWT |
| 41 Hz | <.0001 | GWT |
| 42 Hz | .0002 | GWT |
| 43 Hz | <.0001 | GWT |
| 44 Hz | .0001 | GWT |
| 45 Hz | .0002 | GWT |

Results of genetic variance analysis and kind of estimate applied (GCT: among-twin pair component estimate, GWT: within-pair estimate). REM: rapid eye movement.

* Analysis of variance not applicable (significant differences between the means in monozygotic and dizygotic twins).

MZ_match_: a subgroup of n = 14 MZ twin pairs who were closely matched for age, gender and cohabitation to the group of DZ twins (mean ± SD: MZ_match_: 22.2±2.8yr, 18–27yr, 7m:7f, 10 pairs lived together; DZ: 22.1±2.7yr, 18–26yr, 7m:7f, 10 pairs lived together).

Derivation C3A2.

**Supplementary Figure S6:**


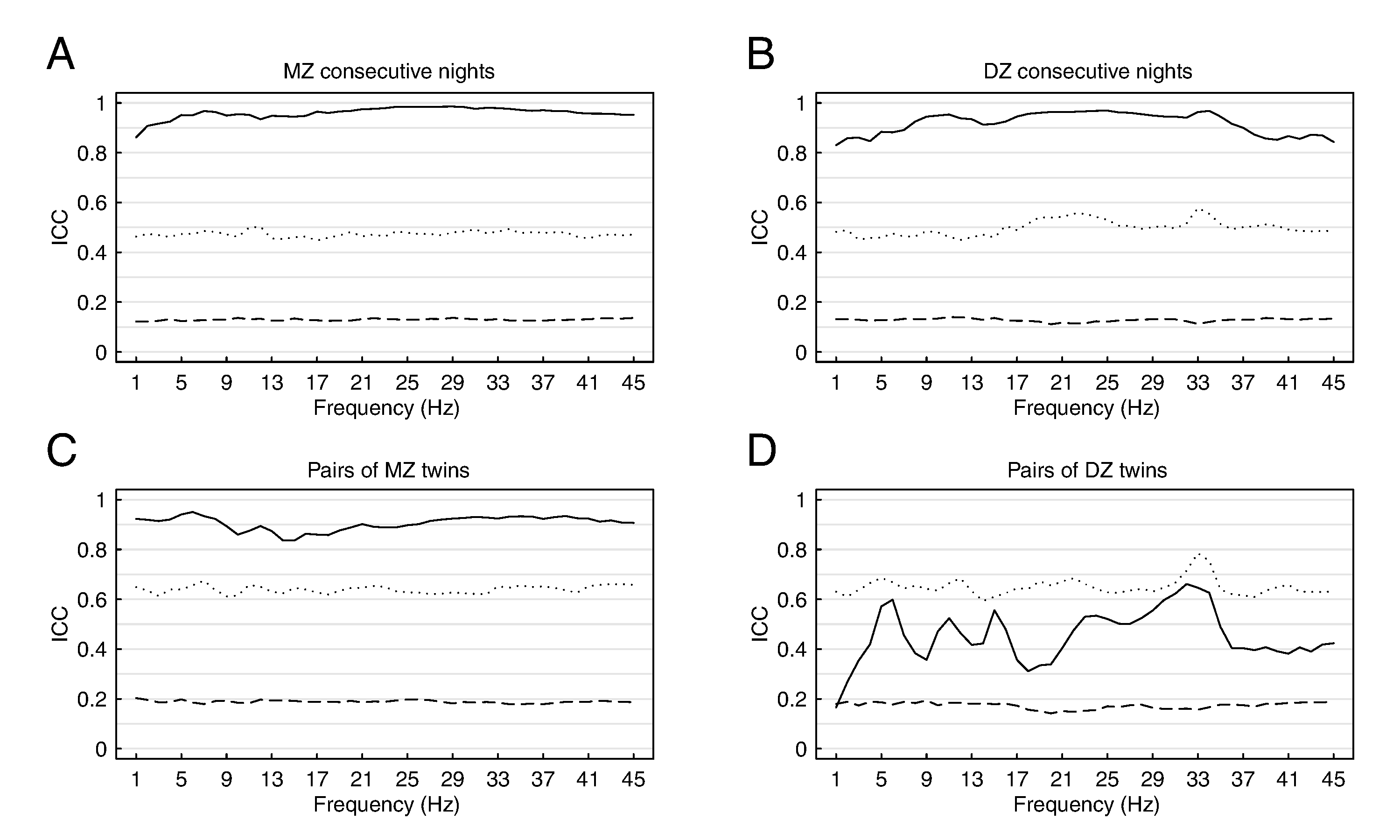
Intraclass correlation coefficients (ICCs) of rapid eye movement (REM) sleep frequency bins from C3A2 EEG derivation. On each plot solid line represents the observed real data, dotted line represents the upper percentile of bootstrapped values and dashed line represents the median of bootstrapped values. (**A**) consecutive nights of each subject in matched monozygotic (MZ_match_) set (n = 28); (**B**) consecutive nights of each subject in dizygotic (DZ) set (n = 28); (**C**) pairs of MZ_match_ twins (each subject represented by a two nights mean, n = 14); (**D**) pairs of DZ twins (each subject represented by a two nights mean, n = 14). On the average, the upper percentile and the median of bootstrapped values differ between groups, which is the outcome of different sample sizes.

**Supplementary Table S7:** Sleep Architecture Parameters Averaged Over Pairs

|  | DZ n = 14 | | | MZ_match_ n = 14 | | |
| --- | --- | --- | --- | --- | --- | --- |
|  | *Night 2* | *Night 3* | *2 nights mean* | *Night 2* | *Night 3* | *2 nights mean* |
| TST | 419.33±6.27 | 423.64±5.65 | 421.49±5.73 | 419.73±6.57 | 415.57±5.67 | 417.65±5.51 |
| SPT | 462.75±4.31 | 466.57±3.23 | 464.66±3.55 | 469.14±1.47 | 469.94±2.69 | 469.54±1.66 |
| SEI | 0.94±0.01 | 0.95±0.01 | 0.95±0.01 | 0.94±0.01 | 0.94±0.01 | 0.94±0.01 |
| SOL | 30.05±4.66 | 27.23±3.55 | 28.64±3.94 | 23.32±2.00 | 21.85±1.76 | 22.58±1.57 |
| non-REM | 324.21±6.17 | 324.89±4.50 | 324.55±5.09 | 323.91±5.64 | 320.92±4.94 | 322.41±5.01 |
| REM | 95.12±4.09 | 98.75±5.07 | 96.93±4.24 | 95.82±4.36 | 94.64±2.57 | 95.23±3.19 |
| RSL | 99.42±9.37 | 99.08±9.19 | 99.25±8.81 | 108.03±9.24 | 100.14±6.95 | 104.08±7.35 |

Group mean ± SEM of sleep characteristics in minutes. TST: Total sleep time, SPT: Sleep period time, SEI: sleep efficiency index, SOL: sleep onset latency, REM: rapid eye movement, non-REM: non-REM sleep duration, REM: REM sleep duration, RSL: REM sleep latency, DZ: dizygotic twins, MZ_match_: matched monozygotic twins.

**Supplementary Table S8:** Genetic Variance Analysis and Intraclass Correlation Coefficients for REM Sleep Architecture and Phasic REM Parameters

| Variable | *P* | GWT vs GCT | ICC MZ_match_ | ICC DZ | ICC MZ_match_ cn | ICC DZ cn |
| --- | --- | --- | --- | --- | --- | --- |
| REM sleep duration | .2848 | GWT | 0.45(0.65, 0.19) | 0.55(0.65, 0.19) | 0.44(0.46, 0.13) | 0.56(0.43, 0.13) |
| REM sleep latency | .5568 | GWT | 0.54(0.69, 0.18) | 0.50(0.72, 0.15) | 0.26(0.50, 0.13) | 0.57(0.53, 0.11) |
| allRD all night | .0021 | GWT | 0.85(0.71, 0.16) | 0.22(0.66, 0.18) | 0.83(0.53, 0.12) | 0.81(0.46, 0.12) |
| 3sRD all night | .0145 | GCT | 0.88(0.79, 0.15) | 0.31(0.66, 0.19) | 0.88(0.59, 0.11) | 0.74(0.48, 0.13) |
| 3sRD 1^st^ cycle | .0022 | GCT | 0.70(0.69, 0.19) | -0.16(0.66, 0.18) | 0.63(0.46, 0.13) | 0.07(0.51, 0.13) |
| 3sRD 2^nd^ cycle | .0101 | GCT | 0.81(0.69, 0.17) | 0.04(0.61, 0.20) | 0.32(0.56, 0.12) | 0.60(0.47, 0.14) |
| 3sRD 3^rd^ cycle | .0201 | GCT | 0.76(0.70, 0.17) | 0.22(0.67, 0.18) | 0.79(0.54, 0.13) | 0.38(0.50, 0.13) |
| 3sRD 1^st^ third | .2366 | GWT | 0.65(0.70, 0.17) | 0.21(0.68, 0.19) | 0.55(0.50, 0.13) | 0.32(0.49, 0.13) |
| 3sRD 2^nd^ third | .0021 | GCT | 0.90(0.78, 0.15) | 0.19(0.62, 0.18) | 0.73(0.52, 0.12) | 0.69(0.46, 0.13) |
| 3sRD 3^rd^ third | .1585 | GWT | 0.67(0.71, 0.17) | 0.31(0.65, 0.20) | 0.61(0.51, 0.13) | 0.44(0.47, 0.13) |
| allRA all night | .0016 | GCT | 0.94(0.88, 0.14) | 0.45(0.66, 0.19) | 0.94(0.71, 0.12) | 0.72(0.48, 0.13) |
| 3sRA all night | .0217 | GCT | 0.91(0.80, 0.16) | 0.50(0.63, 0.19) | 0.93(0.59, 0.12) | 0.78(0.45, 0.13) |
| RinB all night | .0010 | GWT | 0.81(0.70, 0.19) | 0.25(0.66, 0.18) | 0.82(0.51, 0.12) | 0.87(0.49, 0.13) |
| RoutB all night | .2724 | GWT | 0.70(0.67, 0.19) | 0.66(0.62, 0.17) | 0.82(0.50, 0.12) | 0.69(0.47, 0.13) |
| RinB% all night | .0009 | GWT | 0.75(0.63, 0.19) | 0.13(0.70, 0.19) | 0.67(0.46, 0.13) | 0.81(0.48, 0.13) |

Results of genetic variance analysis, kind of estimate applied (GCT: among-twin pair component estimate, GWT: within-pair estimate) and Intraclass Correlation Coefficients (ICCs). REM: rapid eye movement, ICC MZ: ICCs of matched monozygotic (MZ_match_) twins, ICC DZ: ICCs of dizygotic (DZ) twins, ICC MZ_match_ cn: ICCs of consecutive nights for each subject in MZ_match_ group, ICC DZ cn: ICCs of consecutive nights for each subject in DZ group. RA: REM activity, RD: REM density, allRA: the number of all detected REMs, 3sRA: the number of 3-sec mini-epochs containing at least one REM, allRD: allRA / number of REM sleep epochs, 3sRD: 3sRA / number of REM sleep epochs, RinB: the number of all detected REMs inside REM bursts, RoutB: the number of all detected REMs outside REM bursts, RinB%: percentage of REMs in burst state. ICC results include: original sample ICC (upper percentile of bootstrapped data, median of bootstrapped data).

**Supplementary Table S9:** Phasic REM Parameters Averaged Over Pairs

|  | DZ n = 14 | | | MZ_match_ n = 14 | | |
| --- | --- | --- | --- | --- | --- | --- |
|  | *Night 2* | *Night 3* | *2 nights mean* | *Night 2* | *Night 3* | *2 nights mean* |
| allRD all night* | 0.67±0.04 | 0.69±0.03 | 0.68±0.03 | 0.63±0.04 | 0.64±0.04 | 0.64±0.04 |
| 3sRD all night | 2.06±0.19 | 2.11±0.15 | 2.08±0.16 | 1.87±0.23 | 1.97±0.26 | 1.92±0.24 |
| 3sRD 1^st^ cycle | 1.07±0.14 | 1.20±0.15 | 1.13±0.10 | 1.21±0.25 | 1.10±0.18 | 1.16±0.20 |
| 3sRD 2^nd^ cycle | 1.85±0.19 | 1.73±0.17 | 1.79±0.16 | 1.45±0.17 | 1.80±0.35 | 1.62±0.23 |
| 3sRD 3^rd^ cycle | 1.92±0.19 | 2.11±0.22 | 2.01±0.19 | 1.80±0.28 | 1.79± 0.31 | 1.79±0.28 |
| 3sRD 1^st^ third | 1.12± 0.20 | 1.11±0.15 | 1.11±0.15 | 1.11±0.23 | 1.23±0.22 | 1.17±0.22 |
| 3sRD 2^nd^ third | 1.90±0.18 | 2.03±0.19 | 1.97±0.17 | 1.77±0.32 | 1.92±0.32 | 1.84±0.30 |
| 3sRD 3^rd^ third | 2.39±0.22 | 2.34±0.16 | 2.37±0.17 | 2.10±0.22 | 2.23±0.25 | 2.16±0.22 |
| allRA all night | 831.85±118.58 | 860.03±93.90 | 845.94±100.30 | 767.14±192.80 | 763.78±166.21 | 765.46±178.34 |
| 3sRA all night | 402.14±47.36 | 425.00±39.38 | 413.57±40.95 | 370.60±63.69 | 375.92±55.91 | 373.26±59.28 |
| RinB all night* | 2.63±0.09 | 2.69±0.07 | 2.67±0.08 | 2.59±0.08 | 2.60±0.08 | 2.60±0.07 |
| RoutB all night | 214.71±18.38 | 234.67±18.62 | 224.69±17.44 | 203.21±18.03 | 214.67±14.47 | 208.94±15.95 |
| RinB% all night | 0.66±0.02 | 0.67±0.02 | 0.67±0.03 | 0.66±0.02 | 0.65±0.02 | 0.65±0.02 |

Group mean ± SEM. REM: rapid eye movement, DZ: dizygotic twins, MZ_match_: matched monozygotic twins.

Variables abbreviations as in Table S8.

* Logarithm (base 10) transformed data.

**Supplementary Table S10:** Genetic Variance Analysis and Intraclass Correlation Coefficients on Frequency Bands in REM Sleep from C3A2 EEG Derivation

| Variable | *P* | GWT vs GCT | ICC MZ_match_ | ICC DZ | ICC MZ_match_ cn | ICC DZ cn |
| --- | --- | --- | --- | --- | --- | --- |
| δ | .0072 | GCT | 0.92(0.64, 0.19) | 0.27(0.59, 0.18) | 0.93(0.47, 0.13) | 0.87(0.46, 0.13) |
| θ | .0059 | GCT | 0.94(0.63, 0.19) | 0.51(0.66, 0.19) | 0.96(0.47, 0.13) | 0.90(0.46, 0.13) |
| α | .0019 | GWT | 0.88(0.66, 0.19) | 0.40(0.69, 0.19) | 0.95(0.49, 0.13) | 0.95(0.49, 0.13) |
| σ | .0186 | GWT | 0.86(0.68, 0.19) | 0.45(0.64, 0.19) | 0.94(0.48, 0.13) | 0.92(0.50, 0.13) |
| α/σ | .0052 | GWT | 0.88(0.63, 0.19) | 0.52(0.62, 0.19) | 0.94(0.46, 0.13) | 0.95(0.48, 0.13) |
| low σ | .0097 | GWT | 0.87(0.65, 0.18) | 0.41(0.62, 0.19) | 0.94(0.46, 0.13) | 0.93(0.46, 0.13) |
| high σ | .0409 | GWT | 0.84(0.65, 0.19) | 0.53(0.63, 0.18) | 0.94(0.48, 0.13) | 0.92(0.51, 0.13) |
| β1 | .0016 | GWT | 0.88(0.62, 0.19) | 0.41(0.76, 0.17) | 0.97(0.46, 0.14) | 0.97(0.59, 0.12) |
| β2 | .0307 | GWT | 0.92(0.68, 0.19) | 0.62(0.71, 0.17) | 0.98(0.44, 0.13) | 0.96(0.53, 0.12) |
| φ | .0067 | GCT | 0.93(0.65, 0.19) | 0.40(0.61, 0.19) | 0.96(0.46, 0.13) | 0.87(0.47, 0.13) |

Derivation C3A2. Results of genetic variance analysis, kind of estimate applied (GCT: among-twin pair component estimate, GWT: within-pair estimate) and Intraclass Correlation Coefficients (ICCs). REM: rapid eye movement, ICC MZ: ICCs of matched monozygotic (MZ_match_) twins, ICC DZ: ICCs of dizygotic (DZ) twins, ICC MZ_match_ cn: ICCs of consecutive nights for each subject in MZ_match_ group, ICC DZ cn: ICCs of consecutive nights for each subject in DZ group. ICC results include: original sample ICC (upper percentile of bootstrapped data, median of bootstrapped data).

**Supplementary Table S11:** Genetic Variance Analysis on 1-Hz Frequency Bins in REM Sleep from C3A2 EEG Derivation

| Variable | *P* | GWT vs GCT |
| --- | --- | --- |
| 1 Hz | <.0001 | GWT |
| 2 Hz | .0063 | GCT |
| 3 Hz | .0114 | GCT |
| 4 Hz | .0066 | GCT |
| 5 Hz | .0109 | GCT |
| 6 Hz | .0101 | GCT |
| 7 Hz | .0042 | GCT |
| 8 Hz | .0017 | GWT |
| 9 Hz | .0004 | GWT |
| 10 Hz | .0023 | GWT |
| 11 Hz | .0060 | GWT |
| 12 Hz | .0030 | GWT |
| 13 Hz | .0079 | GWT |
| 14 Hz | .0323 | GWT |
| 15 Hz | .0563 | GWT |
| 16 Hz | .0152 | GWT |
| 17 Hz | .0044 | GWT |
| 18 Hz | .0019 | GWT |
| 19 Hz | .0006 | GWT |
| 20 Hz | .0003 | GWT |
| 21 Hz | .0004 | GWT |
| 22 Hz | .0019 | GWT |
| 23 Hz | .0062 | GWT |
| 24 Hz | .0137 | GWT |
| 25 Hz | .0194 | GWT |
| 26 Hz | .0206 | GCT |
| 27 Hz | .0123 | GCT |
| 28 Hz | .0083 | GCT |
| 29 Hz | .0098 | GCT |
| 30 Hz | .0096 | GCT |
| 31 Hz | .0123 | GCT |
| 32 Hz | .0285 | GWT |
| 33 Hz | .0049 | GWT |
| 34 Hz | .0013 | GWT |
| 35 Hz | .0020 | GWT |
| 36 Hz | .0079 | GCT |
| 37 Hz | .0058 | GCT |
| 38 Hz | .0053 | GCT |
| 39 Hz | .0050 | GCT |
| 40 Hz | .0064 | GCT |
| 41 Hz | .0071 | GCT |
| 42 Hz | .0096 | GCT |
| 43 Hz | .0109 | GCT |
| 44 Hz | .0141 | GCT |
| 45 Hz | .0139 | GCT |

Matched MZ sample. Derivation C3A2. Results of genetic variance analysis and kind of estimate applied (GCT: among-twin pair component estimate, GWT: within-pair estimate). REM: rapid eye movement.

**Supplementary Table S12:** EEG Frequency Bands in REM Sleep Averaged Over Pairs from C3A2 EEG Derivation

|  | DZ n = 14 | | | MZ_match_ n = 14 | | |
| --- | --- | --- | --- | --- | --- | --- |
|  | *Night 2* | *Night 3* | *2 nights mean* | *Night 2* | *Night 3* | *2 nights mean* |
| δ | 2.10±0.03 | 2.09±0.03 | 2.10±0.03 | 2.03±0.04 | 2.04±0.04 | 2.04±0.04 |
| θ | 1.48±0.04 | 1.47±0.04 | 1.48±0.04 | 1.47±0.06 | 1.48±0.06 | 1.48±0.06 |
| α | 1.22±0.04 | 1.21±0.04 | 1.21±0.04 | 1.16±0.05 | 1.16±0.05 | 1.16±0.05 |
| σ | 0.79±0.03 | 0.77±0.04 | 0.78±0.03 | 0.73±0.04 | 0.72±0.04 | 0.72±0.04 |
| α/σ | 0.77±0.04 | 0.75±0.04 | 0.76±0.04 | 0.73±0.05 | 0.72±0.05 | 0.73±0.04 |
| low σ | 0.54±0.03 | 0.52±0.04 | 0.53±0.03 | 0.50±0.04 | 0.48±0.04 | 0.49±0.04 |
| high σ | 0.41±0.03 | 0.39±0.04 | 0.40±0.03 | 0.34±0.04 | 0.33±0.04 | 0.34±0.04 |
| β1 | 0.81±0.04 | 0.79±0.04 | 0.80±0.04 | 0.69±0.05 | 0.69±0.05 | 0.69±0.05 |
| β2 | 0.23±0.04 | 0.22±0.04 | 0.22±0.04 | 0.18±0.06 | 0.18±0.05 | 0.18±0.05 |
| φ | -0.25±0.02 | -0.26±0.02 | -0.25±0.02 | -0.29±0.04 | -0.28±0.04 | -0.28±0.04 |

Group mean ± SEM. Logarithm (base 10) transformed mean power densities from C3A2 EEG derivation (given in μV²).

EEG: electroencephalogram, REM: rapid eye movement, DZ: dizygotic twins, MZ_match_: matched monozygotic twins.

**Supplementary Table S13:** EEG 1-Hz Frequency Bins in REM Sleep Averaged Over Pairs from C3A2 EEG Derivation

|  | DZ n = 14 | | | MZ_match_ n = 14 | | |
| --- | --- | --- | --- | --- | --- | --- |
|  | *Night 2* | *Night 3* | *2 nights mean* | *Night 2* | *Night 3* | *2 nights mean* |
| 1 Hz | 1.46±0.03 | 1.47±0.03 | 1.47±0.03 | 1.38±0.04 | 1.39±0.04 | 1.39±0.04 |
| 2 Hz | 1.63±0.03 | 1.62±0.03 | 1.62±0.03 | 1.55±0.04 | 1.56±0.04 | 1.56±0.04 |
| 3 Hz | 1.45±0.03 | 1.43±0.03 | 1.44±0.03 | 1.39±0.04 | 1.40±0.04 | 1.39±0.04 |
| 4 Hz | 1.27±0.03 | 1.25±0.03 | 1.27±0.03 | 1.22±0.05 | 1.22±0.05 | 1.22±0.05 |
| 5 Hz | 1.14±0.04 | 1.12±0.03 | 1.13±0.04 | 1.09±0.05 | 1.09±0.05 | 1.10±0.05 |
| 6 Hz | 1.03±0.04 | 1.02±0.04 | 1.03±0.04 | 1.03±0.06 | 1.03±0.06 | 1.03±0.06 |
| 7 Hz | 0.91±0.04 | 0.89±0.03 | 0.90±0.03 | 0.92±0.06 | 0.92±0.06 | 0.92±0.06 |
| 8 Hz | 0.82±0.04 | 0.80±0.04 | 0.81±0.04 | 0.80±0.06 | 0.80±0.06 | 0.80±0.06 |
| 9 Hz | 0.74±0.05 | 0.73±0.05 | 0.74±0.04 | 0.67±0.05 | 0.68±0.05 | 0.68±0.05 |
| 10 Hz | 0.64±0.05 | 0.63±0.05 | 0.64±0.05 | 0.57±0.05 | 0.57±0.05 | 0.57±0.05 |
| 11 Hz | 0.49±0.04 | 0.48±0.04 | 0.49±0.04 | 0.46±0.05 | 0.45±0.05 | 0.46±0.05 |
| 12 Hz | 0.35±0.04 | 0.34±0.04 | 0.34±0.04 | 0.32±0.04 | 0.31±0.04 | 0.32±0.04 |
| 13 Hz | 0.25±0.03 | 0.23±0.04 | 0.24±0.03 | 0.21±0.04 | 0.20±0.04 | 0.21±0.04 |
| 14 Hz | 0.18±0.03 | 0.16±0.03 | 0.17±0.03 | 0.13±0.04 | 0.12±0.04 | 0.13±0.04 |
| 15 Hz | 0.15±0.03 | 0.12±0.04 | 0.14±0.04 | 0.06±0.04 | 0.05±0.04 | 0.06±0.04 |
| 16 Hz | 0.08±0.03 | 0.06±0.04 | 0.07±0.04 | -0.02±0.04 | -0.03±0.04 | -0.03±0.04 |
| 17 Hz | 0.02±0.03 | -0.00±0.04 | 0.01±0.04 | -0.09±0.04 | -0.10±0.04 | -0.10±0.04 |
| 18 Hz | -0.03±0.04 | -0.05±0.04 | -0.04±0.04 | -0.15±0.04 | -0.15±0.04 | -0.15±0.04 |
| 19 Hz | -0.07±0.04 | -0.09±0.05 | -0.08±0.04 | -0.20±0.05 | -0.20±0.05 | -0.20±0.05 |
| 20 Hz | -0.11±0.04 | -0.13±0.05 | -0.12±0.05 | -0.24±0.05 | -0.25±0.05 | -0.25±0.05 |
| 21 Hz | -0.16±0.04 | -0.18±0.05 | -0.17±0.05 | -0.30±0.05 | -0.30±0.05 | -0.30±0.05 |
| 22 Hz | -0.24±0.05 | -0.26±0.05 | -0.25±0.05 | -0.36±0.05 | -0.36±0.05 | -0.36±0.05 |
| 23 Hz | -0.31±0.05 | -0.33±0.05 | -0.32±0.05 | -0.42±0.05 | -0.42±0.05 | -0.42±0.05 |
| 24 Hz | -0.39±0.04 | -0.41±0.05 | -0.40±0.04 | -0.48±0.05 | -0.49±0.05 | -0.48±0.05 |
| 25 Hz | -0.47±0.04 | -0.48±0.04 | -0.48±0.04 | -0.55±0.06 | -0.55±0.06 | -0.55±0.06 |
| 26 Hz | -0.54±0.04 | -0.55±0.04 | -0.55±0.04 | -0.61±0.06 | -0.61±0.06 | -0.61±0.06 |
| 27 Hz | -0.61±0.04 | -0.62±0.04 | -0.61±0.04 | -0.67±0.06 | -0.67±0.05 | -0.67±0.06 |
| 28 Hz | -0.67±0.03 | -0.68±0.04 | -0.67±0.04 | -0.72±0.06 | -0.72±0.06 | -0.72±0.06 |
| 29 Hz | -0.73±0.03 | -0.74±0.04 | -0.74±0.04 | -0.77±0.06 | -0.78±0.05 | -0.78±0.06 |
| 30 Hz | -0.78±0.03 | -0.80±0.04 | -0.79±0.04 | -0.83±0.06 | -0.83±0.05 | -0.83±0.06 |
| 31 Hz | -0.84±0.03 | -0.86±0.04 | -0.85±0.04 | -0.88±0.06 | -0.88±0.05 | -0.88±0.06 |
| 32 Hz | -0.89±0.04 | -0.91±0.04 | -0.90±0.04 | -0.93±0.06 | -0.93±0.05 | -0.93±0.05 |
| 33 Hz | -0.93±0.04 | -0.94±0.05 | -0.93±0.05 | -0.99±0.05 | -0.99±0.05 | -0.99±0.05 |
| 34 Hz | -0.97±0.04 | -0.99±0.05 | -0.98±0.04 | -1.04±0.05 | -1.03±0.05 | -1.03±0.05 |
| 35 Hz | -1.03±0.03 | -1.04±0.04 | -1.03±0.03 | -1.08±0.05 | -1.08±0.05 | -1.08±0.05 |
| 36 Hz | -1.08±0.03 | -1.09±0.03 | -1.08±0.03 | -1.13±0.05 | -1.12±0.04 | -1.13±0.04 |
| 37 Hz | -1.13±0.03 | -1.14±0.03 | -1.13±0.03 | -1.17±0.04 | -1.17±0.04 | -1.17±0.04 |
| 38 Hz | -1.17±0.03 | -1.19±0.03 | -1.18±0.02 | -1.21±0.04 | -1.21±0.04 | -1.21±0.04 |
| 39 Hz | -1.21±0.02 | -1.23±0.03 | -1.22±0.02 | -1.25±0.04 | -1.25±0.04 | -1.25±0.04 |
| 40 Hz | -1.25±0.02 | -1.26±0.02 | -1.26±0.02 | -1.29±0.04 | -1.28±0.04 | -1.28±0.04 |
| 41 Hz | -1.29±0.02 | -1.30±0.02 | -1.29±0.02 | -1.32±0.04 | -1.31±0.03 | -1.32±0.04 |
| 42 Hz | -1.32±0.02 | -1.33±0.02 | -1.33±0.02 | -1.35±0.04 | -1.35±0.03 | -1.35±0.04 |
| 43 Hz | -1.36±0.02 | -1.36±0.02 | -1.36±0.02 | -1.39±0.04 | -1.38±0.03 | -1.38±0.03 |
| 44 Hz | -1.39±0.02 | -1.40±0.02 | -1.39±0.02 | -1.42±0.04 | -1.41±0.03 | -1.42±0.03 |
| 45 Hz | -1.42±0.02 | -1.43±0.02 | -1.43±0.02 | -1.45±0.04 | -1.45±0.03 | -1.45±0.03 |

Group mean ± SEM. Logarithm (base 10) transformed mean power densities from C3A2 EEG derivation (given in μV²).

EEG: electroencephalogram, REM: rapid eye movement, DZ: dizygotic twins, MZ_match_: matched monozygotic twins.

Whole MZ set. Derivation C4A1:

**Supplementary Figure S14:**


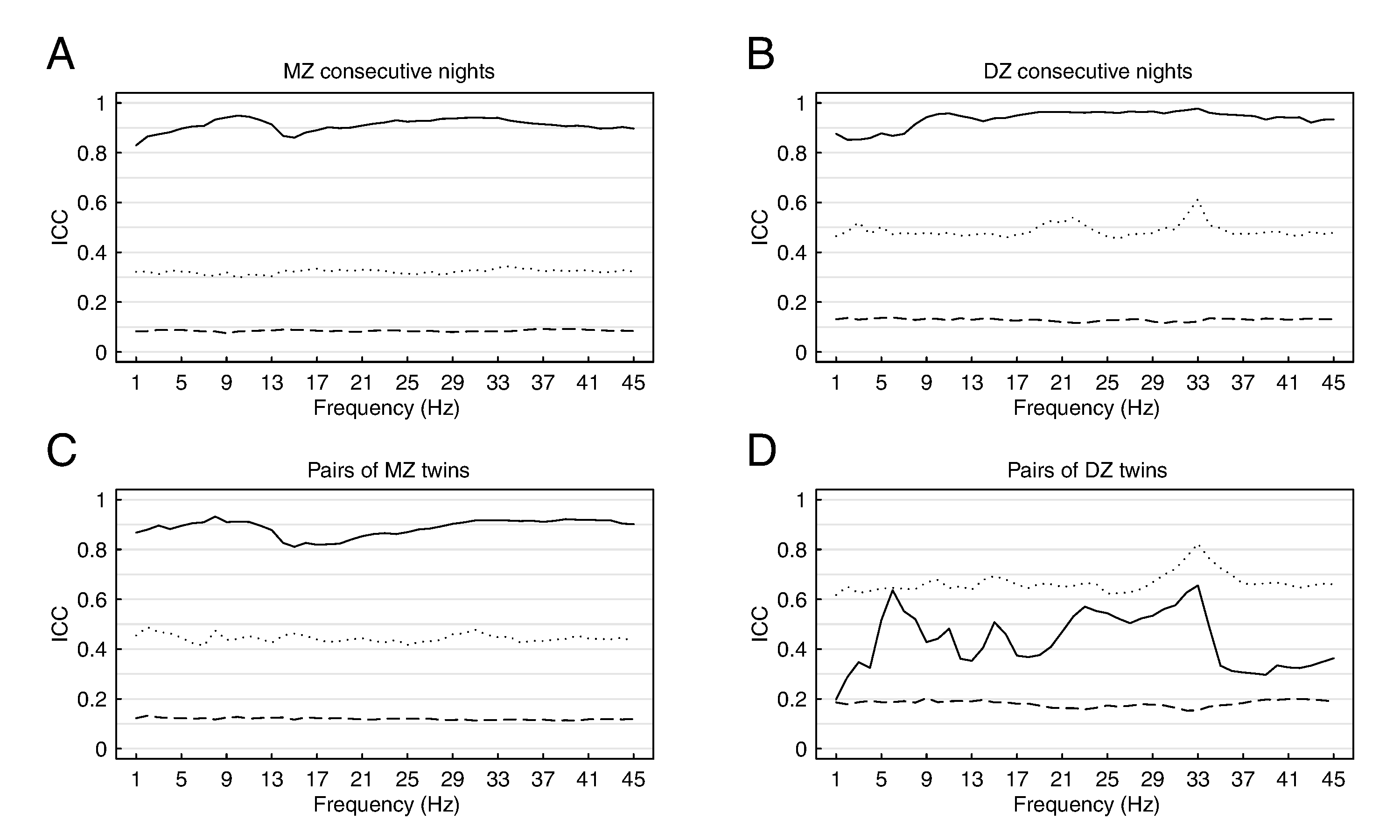
Intraclass correlation coefficients (ICCs) of rapid eye movement (REM) sleep frequency bins from C4A1 EEG derivation. On each plot solid line represents the observed real data, dotted line represents the upper percentile of bootstrapped values and dashed line represents the median of bootstrapped values. (**A**) consecutive nights of each subject in monozygotic (MZ) set (n = 64); (**B**) consecutive nights of each subject in dizygotic (DZ) set (n = 28); (**C**) pairs of MZ twins (each subject represented by a two nights mean, n = 32); (**D**) pairs of DZ twins (each subject represented by a two nights mean, n = 14). On the average, the upper percentile and the median of bootstrapped values differ between groups, which is the outcome of different sample sizes.

**Supplementary Table S15:** Genetic Variance Analysis and Intraclass Correlation Coefficients on Frequency Bands in REM Sleep from C4A1 EEG Derivation

| Variable | *P* | GWT vs GCT | ICC MZ | ICC DZ | ICC MZ cn | ICC DZ cn |
| --- | --- | --- | --- | --- | --- | --- |
| δ | .0005 | GCT | 0.89(0.44, 0.12) | 0.26(0.63, 0.18) | 0.87(0.32, 0.08) | 0.87(0.43, 0.13) |
| θ | .0107 | GWT | 0.91(0.43, 0.12) | 0.61(0.63, 0.19) | 0.90(0.30, 0.09) | 0.88(0.46, 0.13) |
| α | <.0001 | GWT | 0.92(0.43, 0.12) | 0.42(0.67, 0.18) | 0.94(0.33, 0.08) | 0.95(0.49, 0.13) |
| σ | .0005 | GWT | 0.85(0.45, 0.12) | 0.41(0.62, 0.18) | 0.88(0.31, 0.09) | 0.93(0.50, 0.13) |
| α/σ | <.0001 | GWT | 0.91(0.46, 0.13) | 0.45(0.60, 0.18) | 0.94(0.32, 0.09) | 0.95(0.47, 0.13) |
| low σ | .0002 | GWT | 0.87(0.45, 0.11) | 0.36(0.63, 0.19) | 0.90(0.33, 0.09) | 0.93(0.48, 0.13) |
| high σ | .0028 | GWT | 0.82(0.43, 0.12) | 0.49(0.68, 0.19) | 0.86(0.32, 0.08) | 0.93(0.49, 0.13) |
| β1 | .0001 | GWT | 0.84(0.49, 0.13) | 0.46(0.68, 0.18) | 0.90(0.31, 0.08) | 0.96(0.52, 0.13) |
| β2 | .0003 | GWT | 0.90(0.45, 0.12) | 0.57(0.68, 0.17) | 0.93(0.31, 0.08) | 0.96(0.52, 0.12) |
| φ | <.0001 | GWT | 0.92(0.46, 0.12) | 0.32(0.63, 0.19) | 0.90(0.30, 0.08) | 0.94(0.47, 0.13) |

Derivation C4A1. Results of genetic variance analysis, kind of estimate applied (GCT: among-twin pair component estimate, GWT: within-pair estimate) and Intraclass Correlation Coefficients (ICCs). REM: rapid eye movement, ICC MZ: ICCs of monozygotic (MZ) twins, ICC DZ: ICCs of dizygotic (DZ) twins, ICC MZ cn: ICCs of consecutive nights for each subject in MZ group, ICC DZ cn: ICCs of consecutive nights for each subject in DZ group. ICC results include: original sample ICC (upper percentile of bootstrapped data, median of bootstrapped data).

**Supplementary Table S16:** Genetic Variance Analysis on 1-Hz Frequency Bins in REM Sleep from C4A1 EEG Derivation

| Variable | *P* | GWT vs GCT |
| --- | --- | --- |
| 1 Hz | .0014 | GCT |
| 2 Hz | .0008 | GCT |
| 3 Hz | .0007 | GCT |
| 4 Hz | .0005 | GCT |
| 5 Hz | .0036 | GCT |
| 6 Hz | .0193 | GWT |
| 7 Hz | .0033 | GWT |
| 8 Hz | .0050 | GCT |
| 9 Hz | <.0001 | GWT |
| 10 Hz | <.0001 | GWT |
| 11 Hz | <.0001 | GWT |
| 12 Hz | <.0001 | GWT |
| 13 Hz | <.0001 | GWT |
| 14 Hz | .0013 | GWT |
| 15 Hz | .0049 | GWT |
| 16 Hz | .0011 | GWT |
| 17 Hz* | - | - |
| 18 Hz* | - | - |
| 19 Hz* | - | - |
| 20 Hz* | - | - |
| 21 Hz* | - | - |
| 22 Hz | .0001 | GWT |
| 23 Hz | .0004 | GWT |
| 24 Hz | .0006 | GWT |
| 25 Hz | .0007 | GWT |
| 26 Hz | .0005 | GWT |
| 27 Hz | .0005 | GWT |
| 28 Hz | .0006 | GWT |
| 29 Hz | .0007 | GWT |
| 30 Hz | .0006 | GWT |
| 31 Hz | .0002 | GWT |
| 32 Hz | <.0001 | GWT |
| 33 Hz | <.0001 | GWT |
| 34 Hz | <.0001 | GWT |
| 35 Hz | <.0001 | GWT |
| 36 Hz | <.0001 | GWT |
| 37 Hz | <.0001 | GWT |
| 38 Hz | <.0001 | GWT |
| 39 Hz | <.0001 | GWT |
| 40 Hz | <.0001 | GWT |
| 41 Hz | <.0001 | GWT |
| 42 Hz | <.0001 | GWT |
| 43 Hz | <.0001 | GWT |
| 44 Hz | <.0001 | GWT |
| 45 Hz | <.0001 | GWT |

Derivation C4A1. Results of genetic variance analysis and kind of estimate applied (GCT: among-twin pair component estimate, GWT: within-pair estimate). REM: rapid eye movement.

* Analysis of variance not applicable (significant differences between the means in monozygotic and dizygotic twins).
